# P-1051. Antibiotic Elution Patterns Correlate with Antibacterial Activity of Three Antibiotic Antimicrobial Central Venous Catheters (CVCs)

**DOI:** 10.1093/ofid/ofaf695.1246

**Published:** 2026-01-11

**Authors:** Y Lan Truong, Joel Rosenblatt, Bahgat Z Gerges, Ying Jiang, Anne-Marie Chaftari, Ray Y Hachem, Peter Lamie, Dennis Kraus, Issam I Raad

**Affiliations:** UT MD Anderson Cancer Center, Houston, TX; MD Anderson UT, Houston, Texas; MD Anderson UT, Houston, Texas; The University of Texas MD Anderson Cancer Center, Houston, Texas; MD Anderson UT, Houston, Texas; MD Anderson UT, Houston, Texas; UT MD Anderson Cancer Center, Houston, TX; Spectrum Vascular, Denver, Colorado; MD Anderson UT, Houston, Texas

## Abstract

**Background:**

Central Line Associated Bloodstream Infections (CLABSIs) have been designated “never events” hence CVCs with improved antibacterial activities are needed. The FDA recently cleared a new CVC containing Chlorhexidine (C), Minocycline (M) and Rifampin (R). This makes available a third antibiotic containing CVC in addition to MR and CVCs containing C, silver (Si) and the antibiotic Sulfadiazine (Sz). Here, we measured the elution profiles of M, R, and C over time from the three CVCs and assessed how this impacted their antibacterial efficacy and durability.

**Figure:**
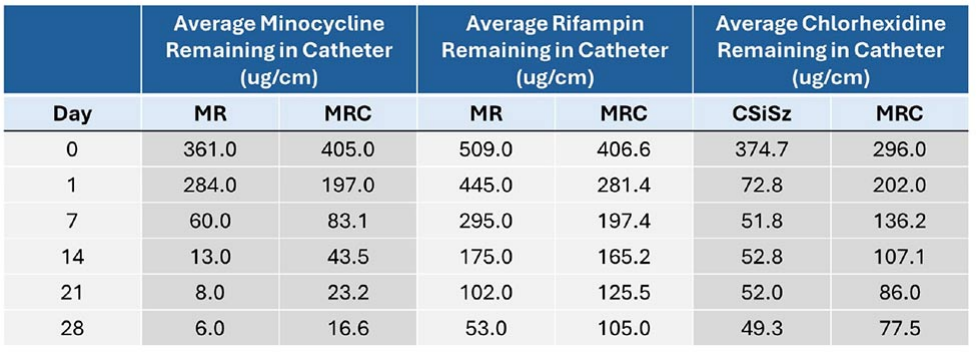
Average Amount of Chlorhexidine, Minocycline, and Rifampin Remaining in the CVCs during Elution

**Figure:**
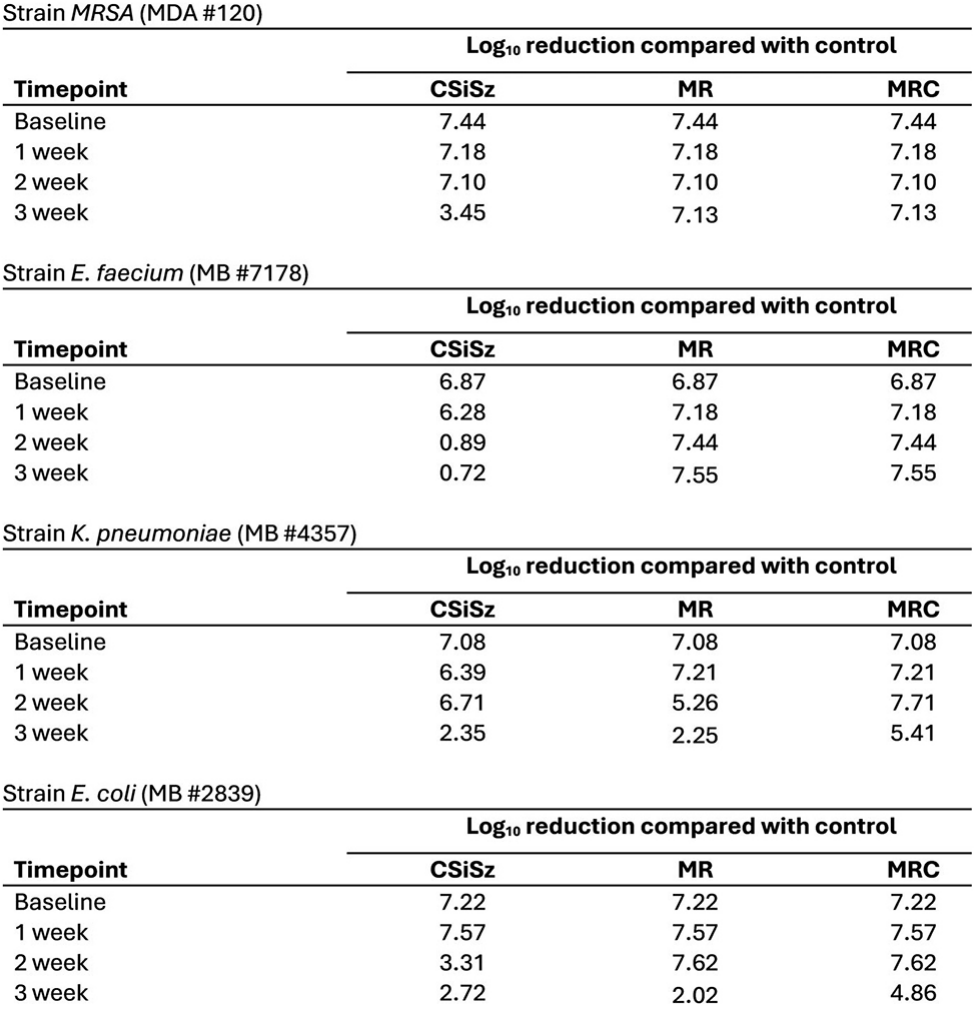
Log Reductions vs Control for MRSA, E. faecium, K. pneumoniae, and E. coli Colonies Recovered from Bacterial Challenges

**Methods:**

MRC CVCs were prepared by a sequential coating process. Commercially available MR and CSiSz CVCs were purchased. CVCs were immersed in plasma for 24 hrs then immersed in serum for 3 weeks. At baseline (24 hrs) and weekly thereafter segments were removed for chemical analysis of C, M and R content remaining by HPLC and for bacterial colonization challenge. Sz elution was not measured as SiSz had very low aqueous solubility. Challenge exposed the segments to inocula of clinical Gram negative and positive CLABSI isolates for 24 hrs following sonication, serial dilution, plating and counted to enumerate viable colonies (CFU/segment). Non-antimicrobial CVCs were controls.

**Figure:**
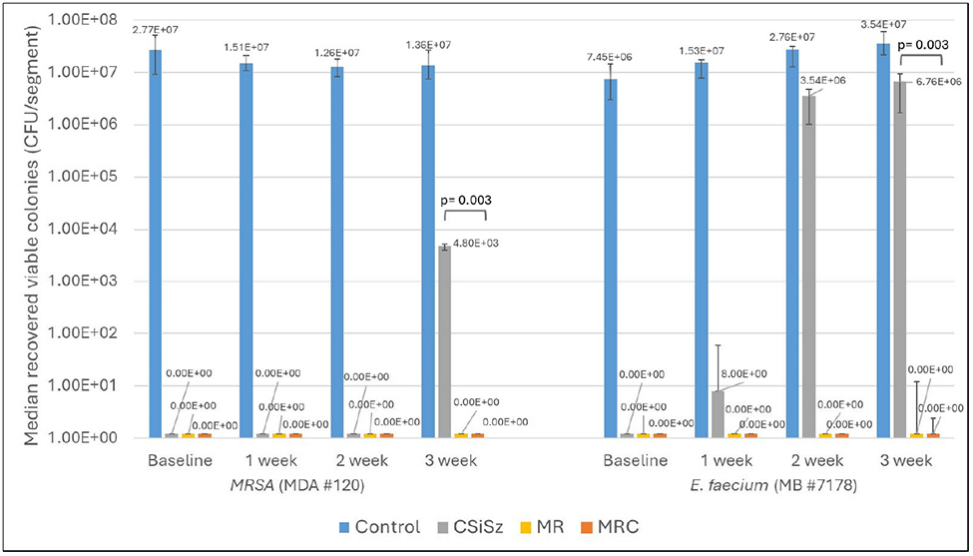
Median Number of CFU/segment Recovered following 24-hr Microbial Challenges of MRSA and E. faecium Statistical comparisons of CSiSz vs MRC CVCs are indicated by horizontal brackets and p-values are indicated above the brackets showing whether differences were statistically significant (if p< 0.05).

**Figure:**
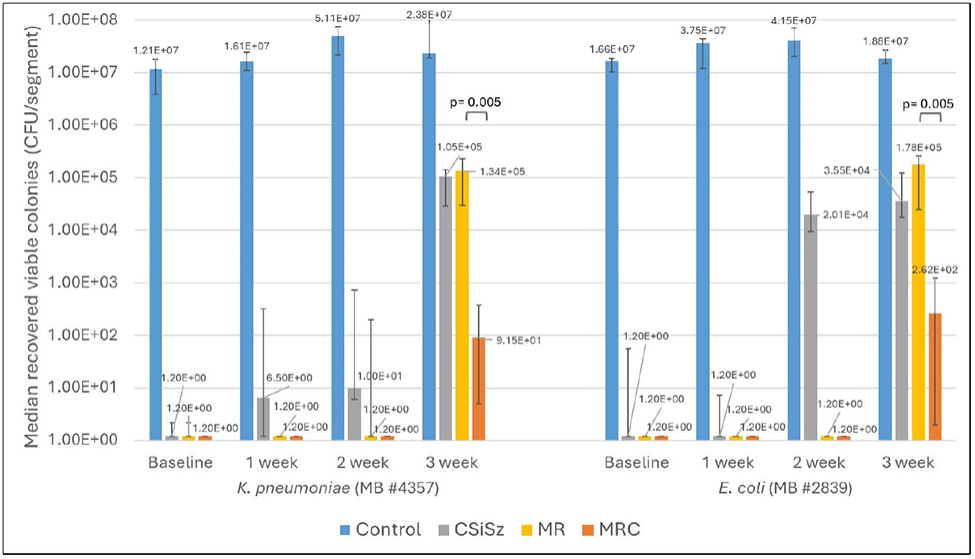
Median Number of CFU/segment Recovered following 24-hr Microbial Challenges of K. pneumoniae and E. coli Statistical comparisons of MR vs MRC CVCs are indicated by horizontal brackets and p-values are indicated above the brackets showing whether differences were statistically significant (if p< 0.05).

**Results:**

Table 1 presents the average amount of C, M and R remaining in the CVCs as they eluted. Table 2 presents log reductions versus control for *MRSA*, *E. faecium*, *K. pneumoniae*, and *E. coli* colonies recovered from bacterial challenges. Figures 1 and 2 present the median number of CFU/segment recovered following 24-hr microbial challenges of *MRSA*, *E. faecium*, *K*. *pneumoniae*, and *E*. *coli*. The CSiSz CVC eluted approximately 80% of its starting C in the first 24 hrs and over 85% after 1 week. The MRC CVC eluted approximately 30% of its initial C at 1 week, 50% at 2 weeks and 55% at 3 weeks. M and R eluted consistently over 3 weeks from both the MR and MRC catheters.

**Conclusion:**

The elution patterns correlate with and explain the limited and early antimicrobial durability of CSiSz against bacteria. Furthermore, based on their slow elution, the MR and MRC CVCs completely prevented colonization by gram positive bacteria through 3 weeks, and the MRC had a significantly greater antimicrobial durability than MR CVC against gram negative bacteria at week 3.

**Disclosures:**

Joel Rosenblatt, PhD, Citius Pharmaceuticals, Inc.: Advisor/Consultant|Citius Pharmaceuticals, Inc.: Grant/Research Support|Citius Pharmaceuticals, Inc.: Patent|Citius Pharmaceuticals, Inc.: Ownership Interest|Citius Pharmaceuticals, Inc.: Stocks/Bonds (Public Company)|Spectrum Vascular: SV Spectrum MRC Central Venous Catheter; SV Spectrum MR Central Venous Catheter; SV Central Venous Catheter|Spectrum Vascular: Ownership Interest Anne-Marie Chaftari, MD, Citius Pharmaceuticals, Inc., Cranford, New Jersey, USA: Grant/Research Support Dennis Kraus, MD, Spectrum Vascular: Patent|Spectrum Vascular: Ownership Interest Issam I. Raad, Distinguished Professor, Citius Pharmaceuticals, Inc. (Grant/Research Support): Advisor/Consultant|Citius Pharmaceuticals, Inc. (Grant/Research Support): Grant/Research Support|Citius Pharmaceuticals, Inc. (Grant/Research Support): Patent|Citius Pharmaceuticals, Inc. (Grant/Research Support): Ownership Interest|Citius Pharmaceuticals, Inc. (Grant/Research Support): Stocks/Bonds (Public Company)|Spectrum Vascular: Patent|Spectrum Vascular: Ownership Interest

